# Protocol to locally express *cxcl12a* during zebrafish olfactory organ development by combining **IR-LEGO** with live imaging

**DOI:** 10.1016/j.xpro.2023.102538

**Published:** 2023-08-23

**Authors:** Marie Zilliox, Vanessa Tillement, Thomas Mangeat, Sophie Polès, Patrick Blader, Julie Batut

**Affiliations:** 1 Unité de biologie Moléculaire, Cellulaire et du Développement (MCD, UMR5077), Centre de Biologie Intégrative (CBI, FR 3743), Université de Toulouse, CNRS, UPS, 118 Route de Narbonne, 31062 Toulouse, France; 2 LITC Core Facility, Centre de Biologie Intégrative (CBI, FR 3743), Université de Toulouse, CNRS, UPS, 118 Route de Narbonne, 31062 Toulouse, France

**Keywords:** Cell Biology, Developmental Biology, Microscopy, Model Organisms, Gene Expression

## Abstract

Temporal and spatial regulation of gene expression is crucial for proper embryonic development. Infrared laser-evoked gene operator (IR-LEGO) can provide information for various developmental processes. Here, we present a protocol to locally express *cxcl12a* during zebrafish olfactory organ development^1^ using a combination of IR-LEGO and live imaging. We describe steps for implementing IR-LEGO, biological sample preparation, live imaging, data collection, and analysis. This protocol can be applied to virtually any genetically modified experimental organism.

## Highlights

• Implementing infrared laser-evoked gene operator (IR-LEGO) for live imaging
• Performing IR-LEGO on live developing zebrafish telencephalon
• Mounting zebrafish embryos to combine IR-LEGO technique with real-time imaging
• Time-lapse of olfactory epithelium morphogenesis using a spinning-disk microscope

## Abstract (editor highlights)

Publisher’s note: Undertaking any experimental protocol requires adherence to local institutional guidelines for laboratory safety and ethics.

## Graphical abstract

**Figure:**
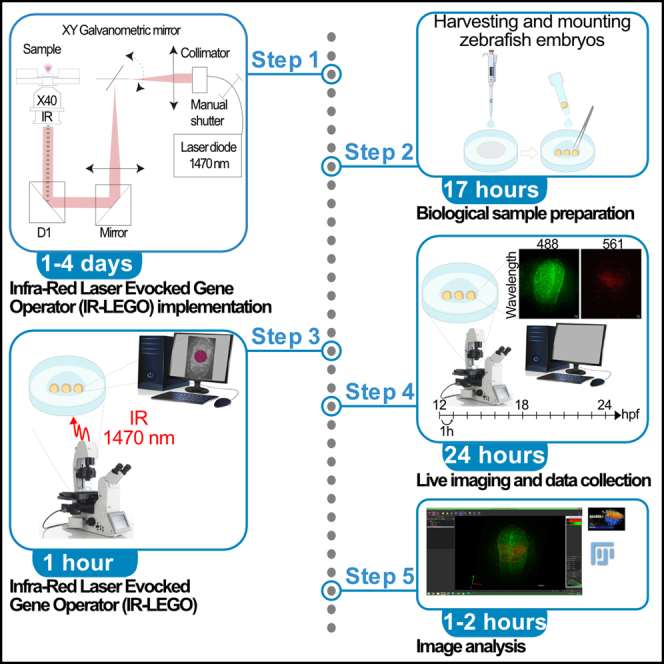

## Before you begin

The protocol below describes a method of gene induction coupled with real-time imaging using stable lines of transgenic zebrafish expressing a gene of interest (here *cxcl12a*) under the control of a heat-sensitive promoter activated by an infrared (IR) laser. One of the main applications of IR-LEGO will be the analysis of gene function *in vivo*, where spatial and temporal control of gene expression are required.^2^^,^^3^ The use of IR-LEGO to induce targeted gene expression can also overcome the problems associated with gene expression in non-specific cells, as it is often the case with other techniques, such as mosaic analysis. Previous work has shown that the Cxcl12a-Cxcr4b chemotaxis signaling pathway is required for the assembly of the olfactory epithelium and the guidance of sensory axons during zebrafish olfactory system development.^1^^,^^4^ In this pathway, the ligand *cxcl12a* (also known as sdf1a) is expressed in the telencephalon as early as 12 hours post fertilization (hpf) and *cxcr4b*, the receptor, is expressed within the early olfactory neurons (EONs).^4^^,^^5^ In embryos mutant for *cxcr4b* or *cxcl12a*, the assembly of the olfactory epithelium is disrupted.^1^ Here, we established a protocol combining the IR-LEGO technique (which allows gene expression to be monitored in time and space, without photodamage) with live imaging, to create a zone of *cxcl12a* expression in the forebrain of zebrafish embryos in order to analyze its effect on olfactory morphogenesis. We first implemented IR-LEGO on a spinning disc confocal system. We then used real-time imaging to analyze the formation of olfactory epithelia following this perturbation. Finally, this protocol was used to rescue genetic mutants with disrupted Cxcl12a signaling and olfactory morphogenesis, in order to restore normal morphogenesis (*Zilliox, Letort* et al.*, in preparation*).

Using the IR-LEGO technique, we aim at targeting the expression of *cxcl12a* within the telencephalon at 12 hpf, and address its effect on the morphogenesis of the olfactory epithelium by live imaging. For this purpose, we use the *Tg(hsp70L:mCherry-cxcl12a)* transgenic line^6^ that expresses *cxcl12a* under the control of a heat shock promoter (hsp70) and the *Tg(-8.0cldnb:lynGFP)* transgenic line^7^ to label the cellular plasma membrane. We crossed these transgenic lines to obtain *Tg(hsp70L:mcherry-cxcl12a); Tg(-8.0cldnb:lynGFP)* double transgenic embryos.

1. The following protocol describes a procedure to trigger gene expression at a specific time and position (i.e., the telencephalon) in the zebrafish embryo using an infrared (IR) laser. A detailed procedure for the implementation of this previously described technique^2^^,^^3^ on a spinning disc is provided to perform real-time live imaging. Here we outline how this protocol may be used to control chemokine gene expression (*cxcl12a*) within the telencephalon of the embryo to study olfactory epithelium morphogenesis.
2. A facility for raising adult zebrafish with the desired genotype and approval for animal experimentations is required to carry out this protocol. The IR-LEGO technique requires a specific transgenic zebrafish line expressing the gene of interest under the control of a heat inducible promoter. Ideally, the gene of interest should be fused with DNA encoding a fluorescent tag or fluorescent protein to allow microscopy monitoring of infrared laser activation during imaging.

### Institutional permissions

All procedures involving zebrafish were carried out in accordance with relevant national and international guidelines and approved by the French veterinary services and the local ethical committee (ID: E31555011, APAPHIS #34368-2021121409357964-v6). Users of this protocol should note that approval for conducting experiments on zebrafish must be acquired in advance from the relevant institutions.

## Key resources table

**Table:** 

REAGENT or RESOURCE	SOURCE	IDENTIFIER
**Experimental models: Organisms/strains**		

Zebrafish: Tg(hsp70L:mCherry-cxcl12a zf3312: zf3312Tg	Wong et al., 2020^6^	ZFIN: ZDB-ALT-211116-7
Zebrafish: Tg(-8.0cldnb:lynGFP) zf106: zf106Tg	Haas and Gilmour, 2006^7^	ZFIN: ZDB-ALT-060919-2

**Chemicals, peptides, and recombinant proteins**		

Microtube 2 mL SafeSeal	Dutscher	Cat#129260
Petri dish 90 × 16.2 mm	Dutscher	Cat#688307
Low-melting agarose type LM-3	Euromedex	Cat#1670
Pronase, PRON-RO	Roche	Cat#10165921001
35 mm dish, high precision 1.5 coverslip 14 mm glass diameter or MatTek dish with over glass	MatTek	Cat#**P35G-0.170-14-C**

**Software and algorithms**		

Metamorph (version 7.10.5.476), Molecular Devices	Leica	Cat#8106710
ROE SysCon (version 1.1.6.9)	Rapp OptoElectronic GmbH	Cat#ROE SysCon Software
Fiji	Fiji/ImageJ2 open source	https://imagej.net/software/fiji/
Imaris	Oxford Instruments	https://imaris.oxinst.com/

**Other**		

External tank for the 1.7 L slope breeding tank	Techniplast	Cat#ZB17BTE
Perforated internal tank for the 1.7 L breeding tank	Techniplast	Cat#ZB17BTISLOP
Polycarbonate lid for the 1.7 L slope breeding tank	Techniplast	Cat#ZB17BTL
Polycarbonate divider for the 1.7 L breeding tank	Techniplast	Cat#ZB17BTD
Glass door enclosure +2/+40°C 140 L	Aqualytic	Cat#438210
Dry bath SBH130DC Stuart	Dutscher	Cat#001184
Dumont#5 forceps – 0.08 × 0.004 mm – carbon steel	Fine Science Tools	Cat#11251-10
Pipetman 20–200 μL - P-200LR	Gilson	Cat#FA10005M
Filter tip 1–200 sterile ClearLine	Dutscher	Cat#014220CL
Pasteur pipette length 230 mm	Dutscher	Cat#020420
Transfer pipette, 3.5 mL, (LxW): 155 × 15 mm, LD-PE, transparent	Sarstedt	Cat#86.1171.001
Egg strainer (tea strainer)	Kitchen Craft	Cat#KCSTRPL70
Rectangular fishnet, fine white mesh 8 cm	Zoomalia	Cat#65284
Stereo zoom microscope SMZ18	Nikon	Cat#MNA53000
Incubator +2°C/+40°C	Fisher Scientific	Cat#15297918
Heat block Stuart SBH130DC model	Dutscher	Cat#001184
Spinning disk - Leica inverted DMi8 microscope	Leica	Cat#11889113
Andor-CSUX1-M1N-5000-4L spinning disk YOKOGAWA CSUX1 M1N1 5000 – rotative disk Nipkow	Leica	Cat#8110529
A fast SCMOS camera (ORCA FLASH4 V2+ Hamamatsu)	Leica	Cat#8110774
Objective HCX PL APO, 40× NA 1.3, WD 0.22	Leica	Cat#11506329
Super Z galvanometric stage (250 μm) for DMi8	Leica	Cat#11640260
Sample holder	Leica	Cat#H301-EC-LG-1x35
IR-LEGO setup (Figure 1A)	Rapp OptoElectronic GmbH	Cat#UGA-42 Geo
DL-1470/1000 Diode laser system Wavelength: 1470 nm/power: 1.0 W	Rapp OptoElectronic GmbH	Cat#DL-1470
Objective HC PL IRAPO 40×/1.10 W CORR	Leica	Cat#15506352
Dichroic D1	Rapp OptoElectronic GmbH	Cat#ZT740/1500
Calibration sample: phosphor micro upconverting particles	Rapp OptoElectronic GmbH (Sundaramoorthy et al.)^8^	https://doi.org/10.1021/acsami.6b15322

## Materials and equipment

**Table:** 0.7% agarose mounting solution

Reagent	Final concentration	Amount
Low melting Agarose	0.7%	0.7 g
1× Fish water	1×	100 mL
**Total**	**N/A**	**100 mL**

The 0.7% low melting agarose in fish water solution can be aliquoted into a 2 mL Eppendorf tube and stored at 4°C for up to 2 months.

**Table:** 1 mg/mL pronase solution

Reagent	Final concentration	Amount
Pronase 10 mg/mL	1 mg/mL	10 mL
1× Fish water	1×	90 mL
**Total**	**N/A**	**100 mL**

Make fresh for same day use. Pronase 10 mg/mL in fish water (stock solution) can be aliquoted and stored at −20°C for one year.

**Table:** Fish water parameters

Reagent	Final concentration	Amount
Alkalinity	0 parts per million (ppm)	N/A
Ammonia	0 ppm	N/A
Hardness	0 ppm	N/A
Nitrite	0 ppm	N/A
pH	5.7	N/A
Phosphate	0 ppm	N/A

Fish water can be sterilized for imaging purposes.

## Step-by-step method details

### Infrared laser induced heating implementation and laser heating theoretical estimation

**Timing: 1 to 4 days**

**Figure 1:**
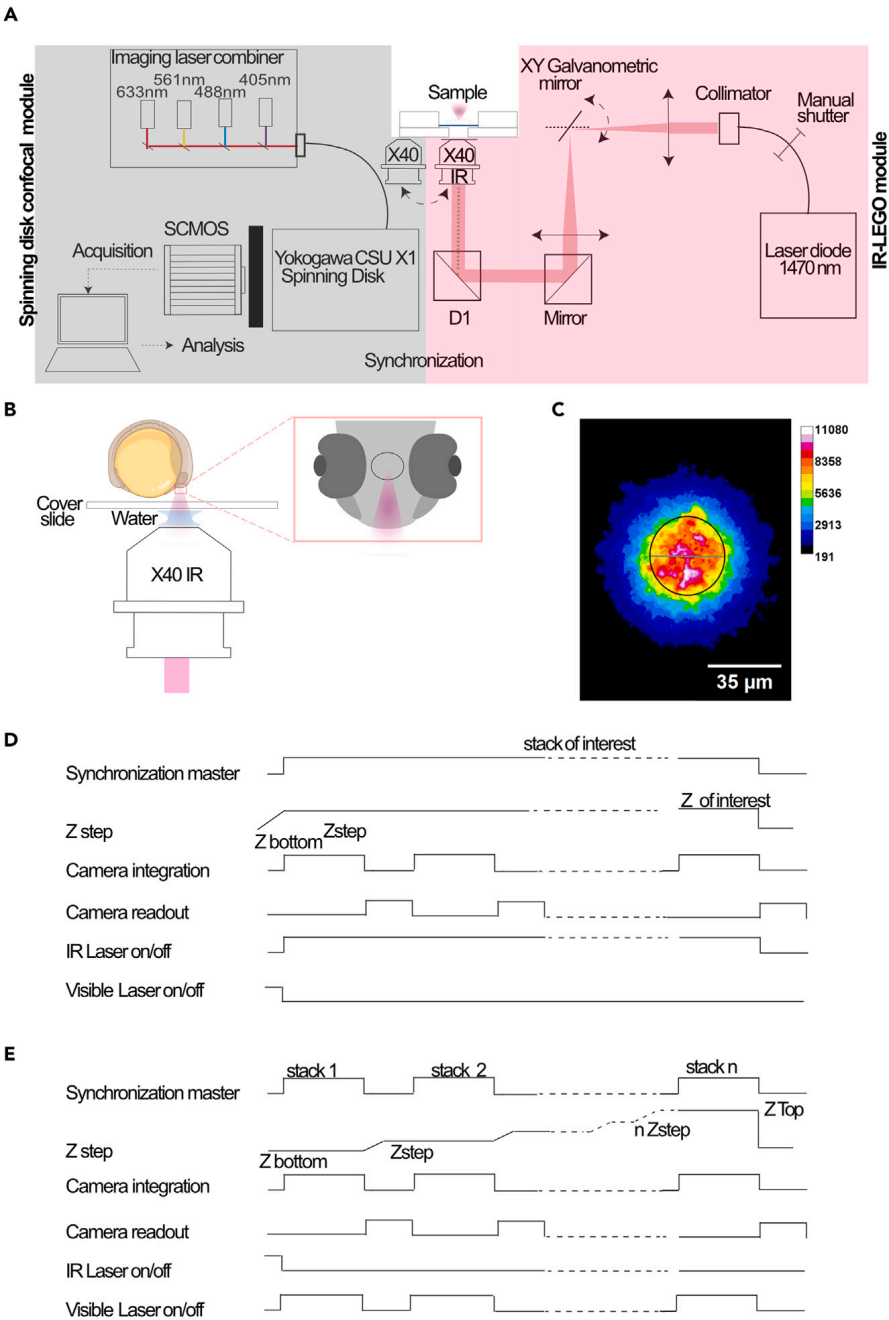
IR-LEGO experimental setup combined with confocal spinning disk for live imaging (A) IR-LEGO system schematic. The infrared photo-irradiation device (IR-LEGO module in light red) is coupled to a spinning disk microscope dedicated to fluorescence live imaging (spinning disk confocal module in gray). IR-LEGO module consists in an infrared laser diode, a galvanometric head mirror to align the laser in the center of the field, a mirror, a dichroic mirror D1 and an IR compatible objective lens, to focalize the laser on the sample. Spinning disk confocal module contains an inverted microscope equipped with an imaging laser combiner composed of 4 diode lasers, a Nipkow spinning head (Yokogawa CSU X1), a fast SCMOS camera and a galvanometric stage for fast image acquisition. (B) A schematic of the sample setup for IR-LEGO using a 40 X APO IR water objective. The magnification shows the laser target in the middle of the zebrafish embryo telencephalon required here for the experiments. (C) IR irradiation of "upconverted phosphor particles" imaged in transmission. "Upconverted phosphor particles" were irradiated with a power of 90% IR laser, inducing an emission wavelength of 545 nm. The irradiation diameter on the calibration sample is estimated to 34 μm (black outlined circle, grey line shows the diameter). (D and E) Experimental system for synchronization of IR irradiation (D) and live imaging (E) conditions. Synchronization timings of all laser-induced heating components followed by (E) imaging chromatogram for each component to reduce phototoxicity. These timings are driven by Metamorph ("synchronization card").

This step describes adaptation of an imaging system (spinning disk) with an infrared laser to irradiate a sample (Figure 1A). The *in vivo* irradiation conditions required to allow expression of a heat shock promoter are described in step 19. The most challenging point is to reduce the cost to obtain a full optically corrected spectral range from 450 to 1470 nm, to combine both live imaging and IR irradiation. There are two ways to implement focalized laser scanning for IR heat. Firstly, we can have a passive optical path (the laser will be in a fixed position at the center of the field of view) with a motorized stage. The second is to use a scanning head (usually consisting of a galvanometric mirror) to change the position of the focused laser.

In both cases, the best way to connect any microscope is to use the corrected optical input at the rear of the microscope without an intermediate optical lens. These lenses are generally optimized for visible light. In fact, it is possible to connect to the tubular lens at the output of the microscope, but generally in the visible range.

1. Use a spinning disk microscope dedicated to confocal fluorescence imaging in the visible range, coupled to an infrared photo-irradiation device (Rapp OptoElectronic GmbH) (Figure 1A).

***Optional:*** A widefield system can be used for smaller samples (bacteria, cell culture, etc…).

2. Couple the IR-LEGO module (Figure 1A, light red rectangle) to the infinity optical corrected input of the inverted microscope (Figure 1A, gray rectangle).

***Note:*** The near infrared laser diode (light source DL-1470/2500/RSP2 Rapp OptoElectronic GmbH) with centered wavelength at 1470 nm leads to a 17°C temperature increase as described in step 3.

***Note:*** Each element from optical fiber to objective lenses, has dedicated optical chromatism correction: fibered collimator, mirror M1, dichroic mirror D1, and dedicated objective lenses (HC PL IR APO 40×/1.10 W CORR).

***Note:*** A galvanometric head mirror (UGA-42 Geo Rapp OptoElectronic GmbH) composed of silver mirror, is conjugated to the back focal plane of the lens to align the laser in the center of the field.

***Note:*** Synchronization between the two modules, IR-LEGO and microscope, during IR irradiation (Figure 1D) or real-time image acquisition (Figure 1E) is performed by the IR laser supplier according to the experimenters’ needs.

***Note:*** IR-LEGO set up (IR laser of 1470 nm) was added on a spinning device to avoid photodamage and photobleaching during live imaging.

***Note:*** Contact your IR laser supplier to check that your microscope is compatible with the addition of a LEGO IR device. They can also help you define what you need to order specifically to add to your spinning disc confocal microscope in order to carry out this protocol.

3. Check *in vivo* (Figure 1B) IR irradiation condition using a dedicated tool (Calibration sample: Phosphor Micro Upconverting Particles, Rapp OptoElectronic GmbH^8^) at the end of the installation, to ensure that the IR-LEGO set up hardware (Figure 1A) is operational.

***Note:*** In our configuration (step 19), using a laser intensity of 90%, we estimate the IR irradiation radius to be approximately 17 μm (Figure 1C). In our experimental setup the laser power in the back pupil plane of the microscope is around 100 mW. This corresponds to a surface irradiation density of 5.5 kW/cm^2^. Hence, in 1 min, the sample receives 330 Kilojoule (KJ). Depending on your biological sample, these values will be adapted. We advise to start with a test experiment depending on the laser power and time of exposure.

***Note:*** With conventional laser (between 100 mW and 200 mW and wavelength between 670 nm and 980 nm compatible in conventional microscopy), the maximal temperature increase with a very high numerical aperture, is only about 5°C. This conclusion is based on the laser-induced heating model developed by Peterman and co-workers.^9^ The only way to achieve an increase from 21 to 37 degrees, and to be able to activate a heat-shock promoter without photodamage, is to increase the wavelength to 1470 nm. Here, we refer to the analytical model introduces by Peterman^9^ to achieve an increase of 17 degrees for 10 cells.

***Note:*** The heat power transferred across two infinitesimally separated concentric cylindrical surfaces with radius, r; is given by the first Equation 1:

(Equation 1) $$q=-K2\pi rL\frac{dT}{dr}$$

where q is the heat power absorbed by water along the distance L of the laser-light path inside the chamber, K is the thermal conductivity of water, r and L respectively the radius and the length of the cylinder, and dT is the temperature difference between the two cylindrical surfaces from the focal point of the laser to the cover slide. By rearranging Equation 1 and integrating dr, the temperature change between two finitely separated cylindrical surfaces can be obtained in Equation 2:

(Equation 2) $$\Delta T\left(r,r^{\prime }\right)=T\left(r\right)-T\left(r^{\prime }\right)=\frac{q}{2\pi KL}\ln\frac{r^{\prime }}{r}$$

Where r' is the radius of the outside cylinder at the surface of cover slide. The absorbed power over the distance L is very small (Pout/Pin close to 1, for L = e, where e is the distance between coverslip end the focalized laser, 170 μm).

***Note:*** Therefore, by using Lambert-Beer’s law, the heat power, q, is given by:

$$q=P_{in}-P_{out}=P_{in}-P_{in}\;\exp\left(-\alpha_{water}e\right)$$

$$q\approx P_{in}\alpha_{water}e$$

$\alpha_{water}$ is the absorption coefficient of water.

r' is equal to 17 μm based on the measurements (Figure 1C).

r, for an over filling of the objective around 30%, is close to e = 170 μm.

By using r equal to 17 μm, r' around 170 μm, Pin(100 mW) $\alpha_{water}$ = 2.81 cm-1 (at λ = 1470 nm and K = 0.60 W.m-1.K-1). Thanks to Equation 2, the difference is around 17°C between the center of the focal plane and the temperature at 21°C on the coverslip.

***Alternatives:*** A measurement using a heat camera to characterize the system experimentally could be envisaged.

### Preparation of experimental zebrafish embryos

**Timing: 17 h**

#### Day 1

Preparation of the desired fish lines for IR-LEGO and live imaging.

4. Embryo production using breeders.

***Note:*** Fishes are maintained at the *Centre de Biologie Intégrative* zebrafish facility in accordance with the rules and protocols in place. The *cxcl12a*^*t30516*^ mutant line has previously been described.^10^

***Note:*** Embryos were obtained through natural crosses and staged according to.^11^

***Alternatives:*** Different transgenic lines with an appropriate genetic background (i.e., gene of interest fused with a fluorescent protein DNA under the control of an hsp promoter) can be used.

**Figure 2:**
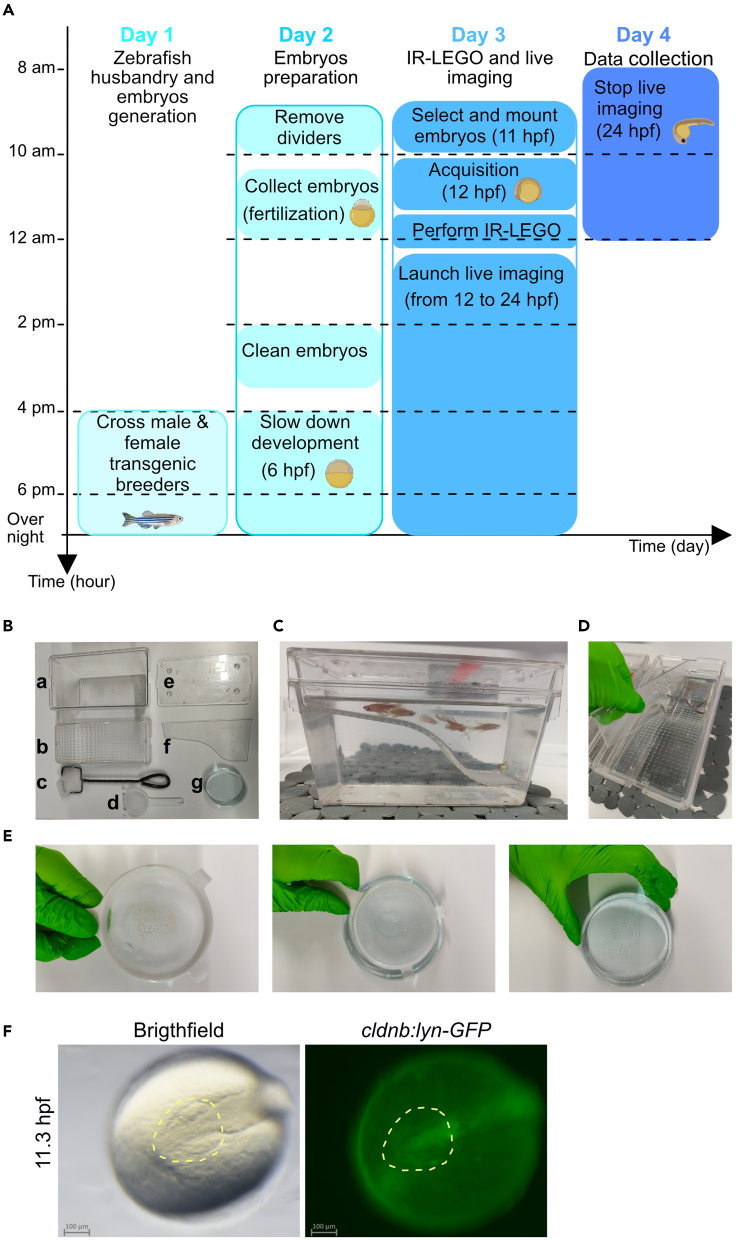
Mating of adult zebrafish and collecting of synchronized embryos (A) Timeline of the 4-day protocol with hours on the y-axis and days in the x-axis. (B) Material used in the fish facility to breed adult zebrafishes and collect embryos. (a) 1.7 L Breeding Tank. (b) Beach breeding compartment. (c) Fishing net. (d) Small strainer. (e) Breeding Tank lid. (f) Divider. (g) Petri dish filled with fish water. (C) Male and female are placed separately on each side of the divider. (D) Remove the divider to start the mating. (E) Collect the embryos with the small strainer (left). Turn the strainer upside down and gently press the embryos in the Petri dish filled with fish water (middle). The embryos are in the Petri dish (right). (F) Frontal and anterior view of the selection of embryos at stage 11.3 hpf in white light (brightfield) and GFP. The anterior part of the head is shown in yellow dotted line.

***Note:*** Handling adult zebrafishes could cause stress which may prevent them from mating. For this reason, breeders are placed in the breeding tanks in the evening before starting the mating process the next morning (see Figure 2A for a timeline overview).

***Note:*** Select the transgenic zebrafish lines whose embryos will be used in the experiment. The transgenic fishes (Tg) breeders used in this study: *Tg(hsp70l:mcherry-cxcl12a)* and *Tg(-8.0cldnb:lynGFP)*.

***Note:*** In the slope breeding tank with a temporary divider and the internal tank with perforated bottom (Figure 2B), gently place the female breeders on one side and the male zebrafishes on the other (Figure 2C).

***Note:*** The number of fish per breeding tank is determined by the size of the tank. We aim for a male: female ratio between 1:2 and 1:3. By placing one male with several females, a higher number of embryos can be harvested the next day. Adjust the number of adults to obtain a sufficient number of embryos the next day, as you will need at least 30 embryos per experiment (maximum number of embryos that could be mounted per experiment which will be selected according to their fluorescence intensity). A couple produces around 150 embryos. For additional information on standard zebrafish husbandry practices, please refer to.^12^

#### Day 2

**Timing: 7 h**

5. Remove the divider between breeders after the light is switched on in the morning to allow males and females to breed (Figure 2D).
  ***Note:*** Depending on the required stage for embryo collection, this step can be delayed and adjusted according to experimental needs. Manipulate the embryos staging by controlling the start of the fish mating through removing the divider between males and females around 10 am (Figure 2A).
  ***Note:*** External fertilization occurs approximately 30 min after removing the divider. The stage of an embryo is referred to by using the acronym hpf (hours post fertilization).
  ***Alternatives:*** Crosses can be performed at any time in the day. However, the next steps should follow the indicated timeline.
  **CRITICAL:** Once you have removed the divider and the adults have laid eggs, you cannot stop the experiment, but you can slightly adjust the timeline (see step number 9 below).
6. 30 min after removing the divider, collect embryos using an egg strainer.
7. Transfer them into a Petri dish filled with fish water (Figure 2E). This ensures a homogeneous staging population of fertilized embryos.
8. Place embryos in a 28.5°C incubator and grow to 11 hpf for IR-LEGO and time lapse imaging (Figure 2A).
  ***Note:*** Dead embryos and unfertilized eggs should be removed.
  ***Note:*** Keep the fish water clean by changing it regularly (at least twice a day) until the embryos have been processed.
  ***Note:*** The optimal density of embryos is 50–100 per 90 mm diameter Petri dish. Use a stereoscopic microscope equipped with a transmitted light source to check the embryos’ state.
  **CRITICAL:** Embryos used for imaging must be handled with care and changed at least once a day with fresh fish water.
  ***Note:*** When the embryo reaches the shield stage i.e., 6 hpf, place the embryos in a 21°C incubator from 4 pm to 10 am to slow down their development and get the stage of interest to perform IR-LEGO the next morning (Figure 2A).
  ***Note:*** Following our timeline, the embryos are at shield stage around 4 pm. At this time, we transfer them into a 21°C incubator to reach the 12 hpf stage the next day at 10 am (Figure 2A).
  **CRITICAL:** Embryos will not develop properly if the temperature is below 21°C. Colder temperatures would lead to high mortality. Do not slow down the embryos before they reach the shield stage because gastrulation has not yet occurred.^11^

### Preparation of agarose Petri dish and mounting zebrafish embryos

#### Day 3

**Timing: 1 h**

9. Sort the proper staged embryos for fluorescent expression under a stereo zoom microscope (SMZ18 Nikon).

***Note:*** At the 4 somite stage (11.3 hpf), selected transgenic claudin [*Tg(cldnb:lyn-GFP)*] embryos based on their green fluorescence (GFP +) in the telencephalon (forebrain, yellow dotted circle, Figure 2F).

***Note:*** Transfer the selected embryos to a Petri dish with fish water.

**Figure 3:**
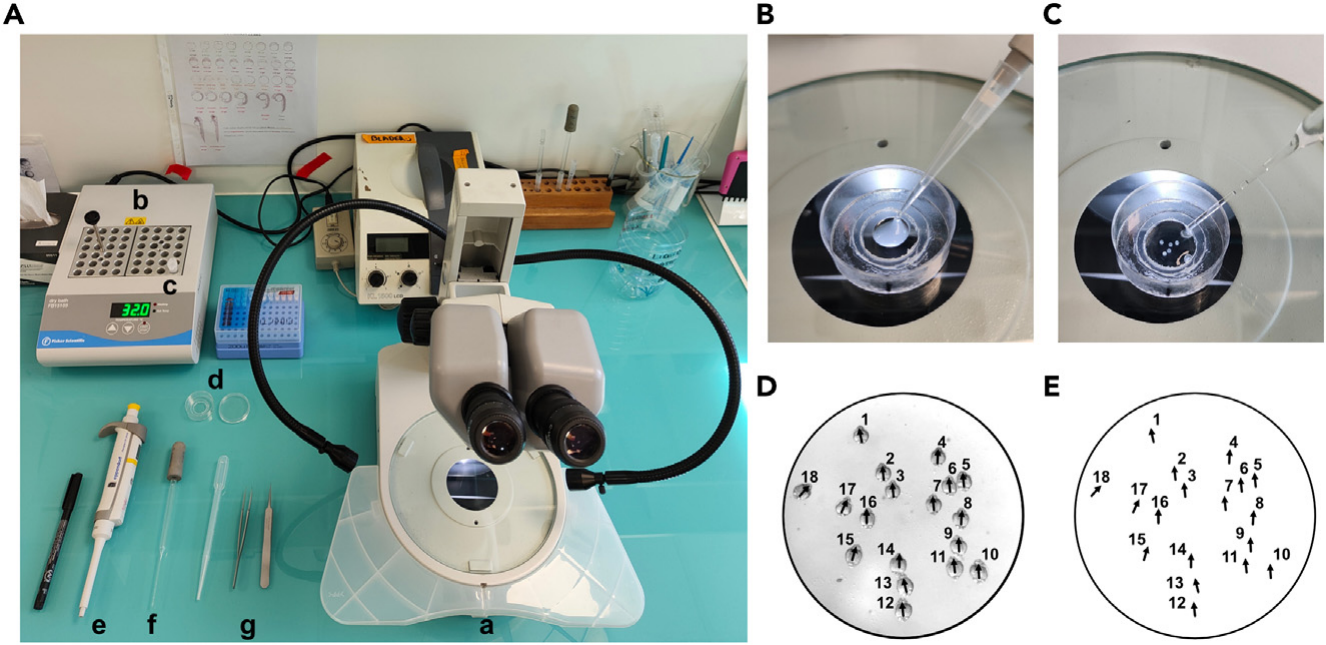
Mounting the zebrafish embryos in 0.7% low melting agarose (A) Material used for mounting embryos. (a) Stereoscopic microscope equipped with a transmitted light source. (b) Heating block set at 32°C. (c) Small aliquot of 0.7% LM agarose in fish water. (d) Small Petri dish. (e) 200 μL pipette. (f) Fine tip glass Pasteur pipet. (g) Dissecting tweezers. (B) Pipet 120 μL of heated 0.7% LM agarose in fish water and drop it in the center of the small Petri dish. (C) Transfer the dechorionated embryos to the agarose drop using a glass Pasteur pipet. (D) Picture showing the orientation of the embryos (black arrowhead points towards the anterior part, the head, of the embryo). (E) Embryos positions are annotated on the lid.

***Note:*** The size of the droplet depends on the size of the glass Petri dish which should be adapted to the microscope setup. We use 120 μL of 0.7% LM agarose on a 35 mm bottom glass Petri dish adapted for an inverted microscope. This system could be adapted to an upright microscope.

***Note:*** As required, place the 2 mL tube containing the 0.7% LM agarose solution (stored at 4°C) in a dry bath (90°C for 15 min) to melt the agarose, then transfer the tube to a dry bath at 32°C for a maximum of 45 min, otherwise the agarose will solidify.

10. At the desired stage (here 12 hpf) remove the chorion under a stereoscopic microscope equipped with a transmitted light source.
  a. Using dissecting tweezers, gently make a tear in the chorion.
  b. Turn it upside down so that the embryo falls out.
    ***Note:*** Following the timeline the embryos reach 12 hpf at 10 a.m., removing the chorion will result in better imaging and allowing the embryos to favorably unwrap itself and elongate during the time-lapse movie.
    ***Alternatives:*** To dechorionate the embryos, a Pronase solution can be used according to “The Zebrafish book” recipes.
    **CRITICAL:** Dechorionated embryos will stick to plastic, therefore, use glass pipettes and glass dishes.
11. Prepare 0.7% low melting (LM) agarose mounting solution. Each 35 mm glass-bottom dish required 1 mL of LM agarose.
  a. Weight out the appropriate amount of LM agarose.
  b. Add sterile fish water and stir to form a homogeneous solution.
  c. Microwave the solution until the LM has completely dissolved in the fish water (10 mL will take about 4 min at maximum power).
  d. Place dissolved LM agarose in a pre-equilibrated 32°C water bath to cool for about 20 min.
12. Add 120 μL of 0.7% LM agarose in fish water heated at 32°C into one glass Petri dish (Figures 3A and 3B).

**CRITICAL:** Be careful to minimize boiling over to reduce evaporation.

***Note:*** When the 4°C aliquoted 0.7% LM agarose is melted do not return it to 4°C but discard what remains if it has not been completely used.

***Note:*** 0.7% LM agarose should be melted at least 45 min prior mounting embryos in order to allow it to melt and adjust to the appropriate temperature in the 32°C dry bath. 0.7% LM agarose in fish water is used to immobilize embryos for live imaging.

**CRITICAL:** Use a traceable thermometer to monitor agarose temperature. Be aware that temperatures above 35°C will activate the heat shock promoter in the whole embryo during mounting. This would cause activation of gene expression throughout the embryo independently of exposure to the IR laser and therefore without spatial control.

13. Use a fine tip glass Pasteur pipet to transfer the embryos in the agarose droplet (Figure 3C). Troubleshooting, problem 1.
14. Before the 0.7% LM agarose solidifies, position the embryos dorsally by using dissecting forceps.

***Note:*** Embryos should be mounted with the somites and trunks at the top and the olfactory system (region of interest, ROI) near the glass slide at the bottom for observation (Figure 3D) on the inverted spinning disk microscope.

**CRITICAL:** There is a short time window of less than 2 min to correctly position the embryos before agarose solidifies. Work quickly but gently during mounting.

15. When all the embryos have been properly oriented, wait for agarose to solidify then fill the whole glass Petri dish glass bottom (homemade device using 35 mm glass Petri dish and 14 mm glass diameter coverslip (Figures 3B and 3C) with 1 mL of 0.7% low melting agarose to obtain a stable and stiff mounting device. Troubleshooting, problem 2.
16. Finally, cover the embryos trapped in the agarose with 2 mL of sterile fish water.
17. Annotate the position of each embryo on the lid of the glass Petri dish (Figure 3E) so that they can later be tracked specifically during live-imaging.

**CRITICAL:** To achieve high resolution imaging, all the embryos should be perfectly oriented the same way when the agarose polymerizes. This way you avoid acquiring thick z-stacks and thus reduce photodamage.

**CRITICAL:** Preparing high quality LM agarose glass Petri dishes filled with fish water is the key step for live imaging of zebrafish embryos.

***Optional:*** Mount one embryo per glass Petri dish to optimize imaging later on.

### IR-LEGO irradiation and imaging settings

**Timing: 3 h**

In zebrafish, EONs are localized as early as 12 hpf at the edge of the neural plate, all around the telencephalon,^5^^,^^13^ and then actively converge from 18 to 24 hpf to form two ellipsoidal neuronal clusters on either side of the telencephalon at 24 hpf. We aim to perform IR-LEGO at 12 hpf to trigger *cxcl12a* expression in the center of the telencephalon. Here, we describe the IR-LEGO irradiation protocol, as well as, how the imaging settings are chosen for real-time imaging. For analysis purposes, the sample is imaged before and after IR irradiation.

**Figure 4:**
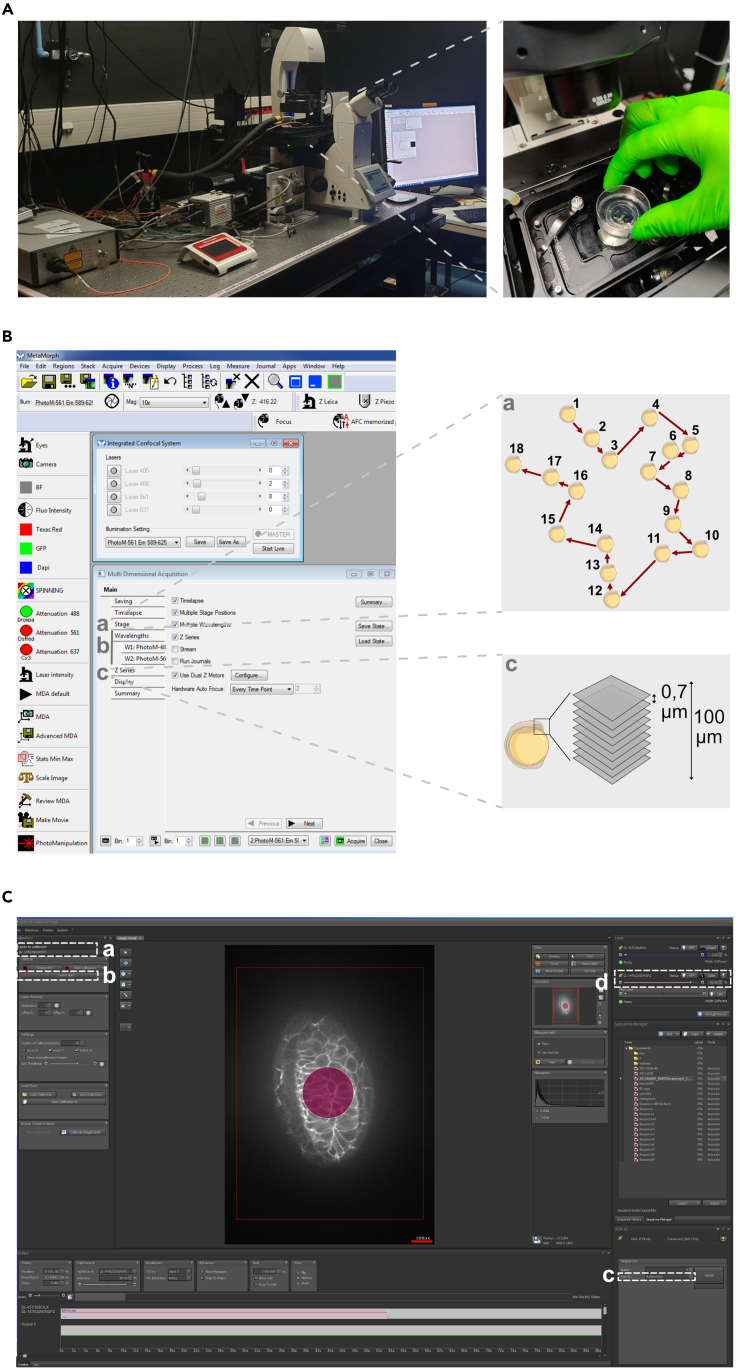
IR-LEGO setup on the Spinning-Disk microscope (A) Placing the sample under the microscope. (B) Metamorph settings. (a) Multi-position pathway; (b) Laser intensity; (c) Z-acquisition scheme. (C) Rapp OptoElectronic settings. (a) Laser 1470 nm; (b) Center the laser; (c) Start of IR irradiation; (d) Adjustment of IR time and intensity.
  d. Use white light to localize the anterior brain of the first embryo. Troubleshooting problem 3.
  e. On Metamorph, set the Z stack. Choose the range of your Z-stack, select “Range around current” and place the stage in the central plane of the Z-stack (Figure 4B).
  f. Choose “Multiposition” and mark the position (Figure 4B).
  g. For every embryo repeat step 18d and 18.f
  h. Set the wavelength parameters to visualize:
    i. In green channel, *Tg(cldnb:lyn-GFP),* the cell membranes. Use an excitation wavelength of 488 nm and a band emission filter 510–540 nm (or other appropriate excitation and emission parameters for the fluorophore). Use a low laser power (∼2% of 150 mW laser, power measured at fiber tip, corresponding to approximately 0.188 mW measured at lens output and 0.368 W/cm^2^ on the sample in our system) and a short exposure time (50 ms) to minimize photobleaching.
    ii. In red channel, *Tg(hsp:mcherry-cxcl12a), mcherry-cxcl12a* expression. Use an excitation wavelength of 561 nm and a band emission filter 589–625 nm (or other appropriate excitation and emission parameter for the fluorophore). Use a low laser power (∼8% of 100 mW laser, power measured at fiber tip, corresponding to approximately 0.55 mW measured at lens output and 1.07 W/cm^2^ on the sample in our system) and an exposure time of 300 ms.
  i. Acquire a stack at both wavelengths with 143 planes every 0.7 μm and automated stages for multiple position acquisition.

***Note:*** Use minimal laser power and exposure time to minimize phototoxicity. We choose to acquire 143 planes at 0.7 μm of each other since the size of the fully formed olfactory epithelium at 24 hpf is around 100 μm. 0.7 μm thickness interval allows us to obtain high quality images. Troubleshooting, problem 2.

18. Set up the noise to signal conditions before using the IR-LEGO technique.^2^^,^^3^ Samples are imaged using an inverted spinning disk confocal microscope (Leica inverted DMi8 microscope, equipped with a CSU-X1 spinning disk head), a sCMOS camera (Hamammatsu Flash4 V2+ camera), and a 40× oil-immersion objective.
  a. Power on the Leica Spinning Disk confocal microscope, the computer and open Metamorph and SysCon (Rapp OptoElectronic GmbH microscopy photomanipulation) softwares. Load the correct journal in the Metamorph software to connect with the SysCon software.
  b. Select the APO 40×/1.3 oil objective.
  c. Place the 35 mm Petri dish with the mounted embryos on the microscope stage. (Figure 4A).

19. Use IR-LEGO to control the timing and pattern of activation of your gene of interest (*mcherry-cxcl12a*).
  ***Note:*** On the Leica microscope control stand, open the IR light path input (manual shutter) and select the right cube, D1 (Figure 1A). Cube D1 is used to reflect the IR laser onto the sample and image the sample using the spinning disk.
  ***Note:*** Check on the stand that the eyepiece port is switched off (100% of the signal sent to the camera port). This shutter is a lock for the IR laser emission (a safety shutter for the laser).
  **CRITICAL:** When eyepiece port is on, the IR laser cannot be used, as it will cause eye damage.
  a. Turn on the IR laser (light source DL-1470/2500/RSP2) and open SysCon software to pilot it.
  b. Select the IR-APO 40×/1.10 W CORR objective (Figure 1A) adapted to IR irradiation.
  c. Within Metamorph software go to the “Multiposition” fold to choose the embryo you want to IR-LEGO (Figures 4A and 4B).
  d. Using claudin-GFP [*Tg(cldnb:lyn-GFP)*] live imaging, manually select the z plane of your choice to be irradiated (here, in the telencephalon center).
  e. Within SysCon software, select the Infrared laser (light source DL-1470/2500/RSP2, Figure 4Ca). Open and load the last Laser calibration folder (Figure 4Cb). Click on “center spot” to put the laser in the middle of the field. Manually choose the ROI corresponding to this laser spot position (using the sequence manager of SysCon software) to see the laser irradiation area on the image of your sample.
    ***Note:*** The laser calibration is dependent on the magnification.
  f. Move your sample to irradiate the desired area (area identified by the displayed ROI). Set the IR laser intensity to 90% in our system (Figures 4C and 4D).
  g. Open the IR shutter and click on the “OFF” Status button to activate (ON) the IR laser (Figures 4C and 4D) for 1 min.
  h. Close the IR shutter and click on the “ON” Status button to inactivate (OFF) the IR laser.
  i. Repeat steps 19.d to 19.h for each embryo to be irradiated.
  j. Close SysCon software, close the IR light path input on the microscope (manual shutter) and switch off the IR laser.
    ***Note:*** We use 90% of the laser power for 1 min. Depending on your biological sample, acquisition system and laser, these values will have to be adjusted. We advise to start with a preliminary experiment to get the optimal setting adapted to your imaging system and your biological sample. The ideal parameters correspond to the minimum power and the minimum exposure time, allowing reproducible activation of the heat-shock promoter to be observed, without inducing detectable cell damage. Troubleshooting, problem 4.
    ***Note:*** In our conditions, we estimate the IR irradiation radius to be approximately 17 μm using a heat-sensitive Phosphor Micro Upconverting Particles provides by Rapp OptoElectronic GmbH (Figure 1C). Troubleshooting, problem 6.
    ***Note:*** This sample can also be used to check the position of the IR laser in the field(i.e., the ROI). Troubleshooting, problem 6.
    ***Note:*** The change of objective between irradiation and imaging, implies a change of immersion medium (water and oil respectively). This is a delicate step if you want to retain the coordinates of the multi-positions, while depositing enough oil on the bottom of the dish to image a large number of samples correctly.
    ***Optional:*** The use of an objective suitable for both irradiation at 1470 nm and imaging could simplify this step.
    **CRITICAL:** IR-Laser is a very dangerous invisible laser that can induce irreversible eye damage, including blindness. At the end of the IR-Laser activation, be sure to close both IR laser shutter (SysCon software) and source. Troubleshooting, problem 5.

### Live imaging of olfactory epithelium morphogenesis using spinning-disk confocal microscopy

**Timing: 24 h**

The early morphogenesis of the olfactory epithelium occurs in the forebrain and starts with the birth of EONs all around the telencephalon at 12 hpf. Between 12 hpf and 18 hpf, EONs complete their initial migration, in which they migrate towards the middle of the telencephalon.^1^ About 6 h later, EONs compact into spheroidal clusters at each side of the telencephalon to form at 24 hpf the olfactory epithelium.^14^ This step describes the live imaging section of the protocol from 12 hpf to 24 hpf.

20. Live imaging of *mCherry-cxcl12a* expression and EONs migration during olfactory epithelium morphogenesis.
  a. Change to the APO 40×/1.3 oil objective for live acquisition.
  b. Repeat steps 19.d to 19.i.
  c. Set time lapse parameters for an automated acquisition of both wavelengths (with 143 planes at 0.7 μm interval and automated stages for multiple positions) every hour during 12 h (Figure 4B).
  d. Launch the 24 h acquisition (Figure 2A).
    ***Note:*** Do not select embryos that are close to each other to image, as this will affect their development and imaging quality.
  e. The next morning, at the end of the time-lapse acquisition, remove your sample and shut down the system.

## Expected outcomes

**Figure 5:**
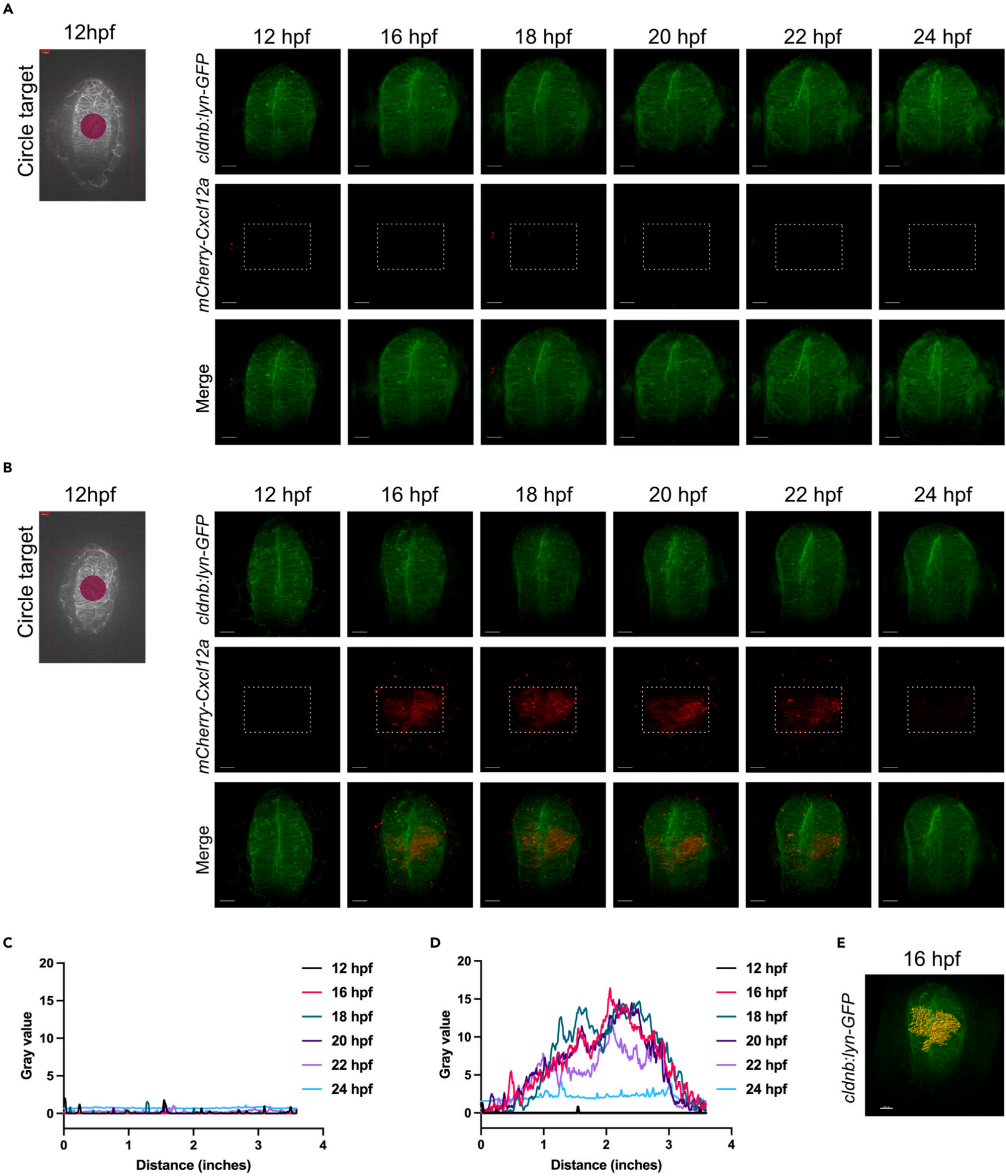
Live imaging after IR irradiation (A and B) Left, image showing the IR laser target area (17 μm radius circle in the middle of the telencephalon) at 12 hpf and Right, imaging over time from 12 hpf to 24 hpf in a (A) *Tg(cldnb:lyn-GFP*), control, or (B) *Tg(cldnb:lyn-GFP)*; *Tg(hsp:mCherry-Cxcl12a)* double transgenic embryos. Scale bar represents 100 μm. (C and D) Plot profiles of mCherry-Cxcl12a expression (Gray value) visualized in (A-B, dashed white rectangle) respectively using Fiji. (E) Visualization of mCherry-Cxcl12a activation at 16 hpf using the Imaris “Surface” tool. The picture in Figure 5B at 16 hpf was used to represent mCherry-Cxcl12a expression. mCherry-Cxcl12a surface is shown in yellow and cldnb:lyn-GFP expression in green.

Using IR-LEGO on live zebrafish embryos, we can control the expression of a gene at a specific time and location. After being born, zebrafish embryos development can be slowed down or accelerated by temperature switches, which makes it easy to control time course experiments. However, managing the spatial activation of a gene is more complicated, thus we propose the IR-LEGO technique. Using this protocol, we can activate a gene of interest by heating a targeted area in the embryo using an IR laser. In a genetically modified embryo mutant for the chemotaxis cytokine Cxcl12a, we activate production of mCherry-Cxcl12a in a 17 μm radius region of interest in the middle of the zebrafish embryos brain at 12 hpf, at the start of the olfactory epithelium morphogenesis. Using *in vivo* live imaging, we are able to visualize the area of mCherry-Cxcl12a activation and follow the olfactory epithelium morphogenesis in response to local expression of the chemotaxis source. We found that mCherry-Cxcl12a activated at 12 hpf is detected from 16 hpf to 22 hpf, with strong expression at 18 hpf (Figure 5).

## Quantification and statistical analysis

### Quantification and visualization of IR-LEGO induced mCherry fluorescence during olfactory epithelium morphogenesis

The live-imaging raw data (.nd) are opened with Fiji and Imaris softwares, analyzed and processed. Live imaging data analysis reveals *mCherry-Cxcl12a* activation area (dotted white rectangle in Figure 5B) after IR irradiation. Embryos lacking the transgene with the heat-sensitive promoter enabling mCherry-Cxcl12a expression serve as negative controls (Figure 5A), as do embryos possessing the transgene imaged prior to IR Laser irradiation (Figure 5B, 12 hpf). Quantification of mCherry intensity without (Figure 5C) or with the *Tg(hsp:mCherry-Cxcl12a)* transgene (Figure 5D) and after IR activation will serve as IR activation control. Our results indicate a 15-fold increase in the expression of the transgene carrying a heat-sensitive promoter after IR irradiation and maximum expression between 16 and 18 hpf, i.e., around 5 h after irradiation (compared Figures 5C and 5D).

1. Quantification of irradiated control embryos using Fiji (Figure 5C). a. Open the first picture (12 hpf, control sample without *Tg(hsp:mCherry-Cxcl12a),* (Figure 5A) with Fiji. b. Define and save a ROI (dotted white rectangle) using the “ROI Manager” Tools. c. Analyze the “Plot Profile” of the defined ROI using “Analyze” and “Plot Profile”. d. For each picture (16–18 – 20–22 and 24 hpf) repeat steps 1.a to 1.c. e. Plot the data obtained (gray value) as a function of distance within the ROI (Figure 5C).
2. Quantification of *Tg(hsp:mCherry-Cxcl12a*) irradiated embryos (Figure 5D).
  a. Perform steps 1.a to 1.d for *Tg(hsp:mCherry-Cxcl12a)* pictures.
  b. Plot the data obtained (gray value) as a function of distance within the ROI (Figure 5D).
3. Visualization of live imaging data reveals *mCherry-Cxcl12a* activation area at 16 hpf (Figure 5E).
  a. With Imaris, open red wavelength (mCherry-Cxcl12a).
  b. Add Channel to open the second green wavelength (cldnb:lyn-GFP).
  c. Adapt the fluorescence signal to visualize structures of interest.
  d. Open “Surface” to quantify mCherry-Cxcl12a expression.
  e. Select the red channel.
  f. Follow Imaris software instructions.

***Note:*** We use the Imaris 8.3 | Release Notes - Imaris - Oxford Instruments (oxinst.com). The parameters of the “Surface” option for quantifying and visualizing the area of interest, here mCherry-Cxcl12a expression, are experiment-specific and can be adjusted by following the Imaris software developer’s instructions.

***Note:*** To further enhance the analysis, Imaris offers the possibility of exporting data related to the surfaces created, with various parameters such as area, sphericity, intensity, volume and their positioning in X, Y and Z as a function of the reference frame.

**CRITICAL:** To ensure consistent analysis, make sure that the quantification parameters for the “Surface” option are the same throughout the analysis.

### Quantitative quality control

For quantitative quality control of an IR activated gene expression, we perform a complete heat shock on our embryos. Indeed, in this protocol we indicate to mount *Tg(hsp:mCherry-Cxcl12a)* embryos in low melting 0.7% fish water agarose which must first be melted at 80°C and then reduced and maintained at 32°C. Thus, to ensure that the mCherry signal observed in the experiment is due to the activation of the IR laser and is not a consequence of the mounting, we place in parallel embryos in a water bath at 38°C in order to activate a total heat shock of *Tg(hsp:mCherry-Cxcl12a)*.

## Limitations

The minimal size of the IR laser beam could still be too big to accurately target the area of interest. In our device the minimum radius is 17 μm. For instance, the IR laser must be able to irradiate a user-defined region. The size of the region can be modified with objectives of different magnification. However, these objectives must be IR-compatible and suitable for the experiment conditions, which is not always the case.

Because the IR laser induces heat, we cannot exclude heat diffusion within the sample. Depending on the position of your tissue in the embryo, this diffusion will be more important along the z axis, the further your tissue is from the IR source. Indeed, this diffusion could interfere with your experiments by preventing tight spatial targeting of expression.

Each transgene (heat-sensitive promoter controlling the expression of a fluorescent protein fused to your gene of interest) will have its own expression kinetics. Here, activation of the *Tg(hsp:mCherry-Cxcl12a)* transgene at 12 hpf induces expression at 16 hpf, which peaks at 18 hpf and then decreases at 20 hpf and 22 hpf to die out at 24 hpf. This represents approximately 6 h of expression. This timing may not be compatible with all experiments.

## Troubleshooting

### Problem 1 (from step 10)

During acquisition, the live embryos can move or dislodge from the agarose.

### Potential solution

Movement of the zebrafish between 12 hpf and 24 hpf may be caused primarily by failure of the mounting medium or embryo growth. One solution may be to ensure that the embryo is well covered by the mounting medium by adding more mounting medium. During the mounting step, adding the embryos can dilute the agarose. This problem may be solved by:

• Reducing the amount of pipetted water to the maximum.
• Increasing the concentration of agarose to 0.9% (do not exceed 1% as this may cause developmental issue).

### Problem 2 (from step 12)

Developmental problems occur or the embryo shows signs of apoptosis during the experiment.

### Potential solution

Consider limiting phototoxicity by using lower laser power, exposure and acquisition time.

### Problem 3 (step 16)

Finding the region to irradiate with the IR corrected objective.

### Potential solution

Training to recognize your region to be activated in transmitted light may be required with the IR corrected objective to facilitate the procedure.

### Problem 4 (steps 15)

Diffusion of the heat-sensitive promoter in depth (z dimension).

### Potential solution

To limit z-diffusion, one solution is to irradiate in a more superficial plane to anticipate diffusion. Another possibility is to adapt irradiation time and/or IR laser power, to reduce the activation zone and thus diffusion. These parameters can be adjusted according to the needs of the experiment.

This problem may be caused by z-light diffusion. It may be important to quantify this with a fluorescent reporter as indicated from step 15.

### Problem 5 (step 15)

Infrared laser can cause serious damage to the eye.

### Potential solution

Once the samples are placed on the microscope stage, the selection of the regions of interest should be done here through the digital interface provided by MetaMorph or Syscon, allowing to perform the whole procedure without looking directly at the sample through the oculars.

Make sure to check that the IR-LEGO shutter is on and that the IR laser activation is 0% off before using the microscope eyepieces.

### Problem 6 (steps 15)

There is no visible expression of mCherry-Cxcl12a*,* i.e., no activation of the heat shock sensitive promoter.

### Potential solution

This problem may be resolved by checking the alignment, focus and power of the IR laser frequently (your supplier will be able to assist you).

• The spatial calibration of the IR laser in x, y positions can be checked using a calibration sample supplied by Rapp OptoElectronic GmbH. This sample consists in heat-sensitive Phosphor Micro Upconverting Particles with a conversion emission wavelength of 545 nm. The position of the laser can thus be indirectly "imaged" and verified.
• The focus of the laser can be checked and adjusted using the same sample (signal maximization).
• The laser power at the lens entrance (unfocused laser) can be checked using a power meter with an appropriate probe.
• If required, a regular check of the laser power can be performed just before sample irradiation using the calibration sample (check of the signal intensity under identical experimental acquisition conditions). Similarly, a rapid functional test on a biological sample can be set up (example: cell death test, 2 min–90% in our system).

Another way of solving the problem would be to perform a total heat shock by incubating the embryos in a 37°C water bath for 20 min and visualizing the expression of the fluorescent protein coupled to the gene of interest (here mCherry-Cxcl12a).

## Resource availability

### Lead contact

Further information and requests for resources and reagents should be directed to and will be fulfilled by the lead contact, Julie Batut (julie.batut@univ-tlse3.fr).

### Materials availability

This study did not generate new unique reagents.

## Data Availability

This study did not generate/analyze new datasets/code.
